# Successful treatment of refractory cutaneous Crohn’s disease with upadacitinib: A case report

**DOI:** 10.1016/j.jdcr.2026.05.061

**Published:** 2026-06-03

**Authors:** Christiaan H. Noot, Emily P. Beck, Jennie T. Clarke, Zachary H. Hopkins

**Affiliations:** a University of Utah Spencer F. Eccles School of Medicine, Salt Lake City, Utah; b Department of Dermatology, Autoimmune Skin Diseases Clinic, University of Utah Spencer F. Eccles School of Medicine, Salt Lake City, Utah

**Keywords:** CCD, cutaneous Crohn’s disease, dermatology, IBD, inflammatory bowel disease, JAK inhibitor, upadacitinib

## Introduction/Background

Crohn’s disease (CD) is a chronic granulomatous inflammatory disorder that primarily affects the gastrointestinal (GI) tract. However, up to 44% of patients experience extraintestinal manifestations, including cutaneous.^1^ Cutaneous Crohn’s disease (CCD) frequently occurs at GI-tract adjacent sites (external genitalia, mouth/lips) but can involve noncontiguous, or metastatic sites as well.^2^ CCD is polymorphous, manifesting as plaques, nodules, ulcers, or fissures and can mimic hidradenitis suppurativa (HS), pyoderma gangrenosum, or other neutrophilic dermatoses.^2^ CCD without GI involvement is rare but has been reported.^3^, ^4^, ^5^ CCD treatment is challenging and includes immunomodulatory therapies such as corticosteroids, azathioprine, and anti–tumor necrosis factor agents.^2^ More recently, upadacitinib, an oral Janus kinase inhibitor (JAKi) selective for JAK1 was approved for CD.^6^ However, literature on JAKi efficacy in isolated CCD remains limited, with only 1 previous case reported.^7^ Herein, we report a case of two-fold importance: (1) a rare instance of CCD without GI involvement and (2) effective treatment of recalcitrant disease with upadacitinib.

## Case report

**Fig 1:**
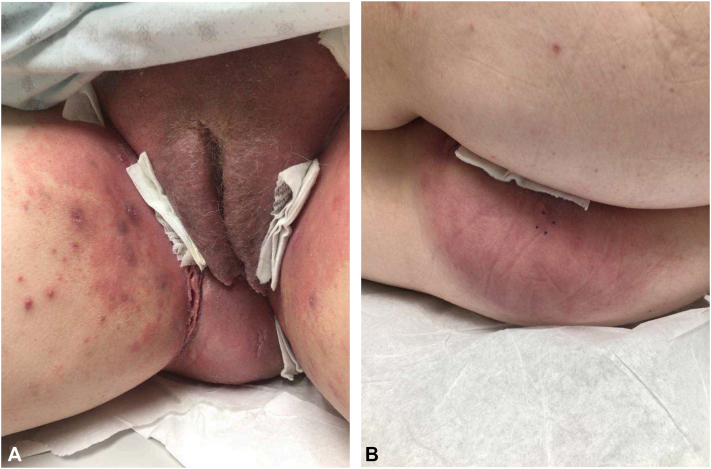
Chronic Crohn’s disease showing erosions and knife-like fissures on the vulva and in skin folds **(A)**, as well as recurrent perianal indurated plaques **(B)**.

**Fig 2:**
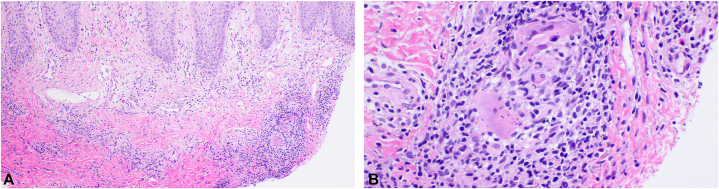
Cutaneous biopsy from the perianal region at 100× **(A)** and 400× **(B)** magnification demonstrating acanthosis, spongiosis, and focal granulomatous dermatitis.

**Fig 3:**
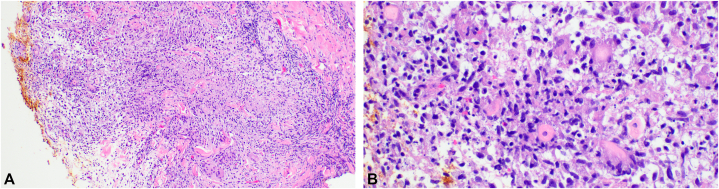
Cutaneous biopsy from the left lateral buttock at 100× **(A)** and 400× **(B)** magnification demonstrating granulomatous dermatitis with mixed inflammation and spongiosis.

**Fig 4:**
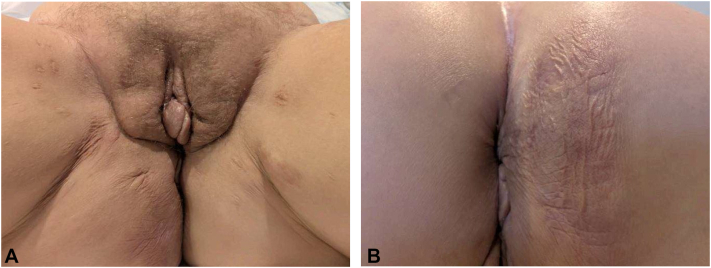
Resolved vulvar fissures and erosions **(A)**, and softening of indurated scarred plaques in the gluteal cleft **(B)** after 6 months of therapy with upadacitinib 30 mg daily.

A woman in her 30’s presented with recurrent perianal fistulas, painful erosions of the vulva, and indurated plaques of the gluteal cleft (Fig 1, *A* and *B*). She had no history or present findings in the axillae, inframammary folds, or abdomen. Cultures were negative, and biopsies revealed a deep granulomatous dermatitis consisting of epithelioid noncaseating granulomas concerning for CCD (Figs 2 and 3). Other etiologies were considered, such as HS and granulomatous drug eruption, but morphology (lack of abscesses, comedones, etc), limited distribution, and histology best supported CCD. Colonoscopy, upper endoscopy, and capsule endoscopy showed no evidence of CD. However, given the clinical morphology and supportive histology, CCD without GI involvement was favored. Adalimumab and ustekinumab were each trialed for 5 months consecutively with initial improvement, but recurrent flares required frequent prednisone tapers. Subsequently, monthly infliximab 10 mg/kg was started alongside methotrexate, topical tacrolimus, and intralesional triamcinolone. She experienced significant improvement, but breakthrough flares necessitated frequent intralesional triamcinolone injections. Additionally, migraine flares and fatigue coincided with infusions and methotrexate doses. Infliximab was stopped after 2 years of treatment and systemic therapy was subsequently transitioned to upadacitinib 15 mg daily. Within 1 month, she reported significantly decreased pain, swelling, and drainage. At 3 months, she reported near complete resolution of fissures and erosions with no active inflammation – but occasional flares. She also reported much improved energy and quality of life. To further decrease continued occasional flares, upadacitinib was increased to 30 mg daily. After 6 months of 30 mg daily, she remains in complete remission without flares and continued softening of indurated scarred plaques (Fig 4, *A* and *B*). She has had no adverse events from the upadacitinib to date.

## Discussion

HS and CCD can have significant clinical overlap, including nodules, abscesses, sinus tracts, and localization in intertriginous areas.^8^ In cases with recurrent fistulas, it is always important to consider HS as a possible primary or concomitant etiology. Several features help differentiate HS and CCD in this patient. The localization of disease to the inguinal and perianal regions, with no prior or subsequent involvement of other intertriginous areas such as the axillae, inframammary, or abdominal folds, makes HS less likely. Clinically, the lack of draining abscesses and comedones further supports this differentiation. Lastly, the presence of deep dermal characteristic granulomas, a histologic finding rarely observed in HS, supports a CCD diagnosis when paired with the clinical presentation.^9^

CCD can be severely debilitating and is frequently resistant to conventional therapies. Anti-tumor necrosis factor agents remain first-line, with ustekinumab, interleukin-23 inhibitors, and vedolizumab as alternatives; however, some patients fail to respond or cannot tolerate these agents. Upadacitinib is FDA-approved for CD, ulcerative colitis, atopic dermatitis, and rheumatoid arthritis. Its role in CCD remains poorly defined, but this case highlights an example of rapid disease improvement and long-term clearance of CCD with JAK-STAT inhibition. Mechanistically, IL-6 and IFN-γ, proinflammatory cytokines downstream of the JAK-STAT pathway, are implicated in CD.^10^ Upadacitinib inhibits these pathways via selective JAK1 inhibition.

This represents both a rare and challenging case of CCD without GI involvement successfully treated with upadacitinib. Further investigation is needed for the use of JAKi in treating extraintestinal inflammatory bowel disease.

## Conflicts of interest

Dr Hopkins receives consulting fees from Priovant Therapeutics, Dren Bio, and is supported by a career development award from the Dermatology Foundation. Authors Noot, Beck, and Dr Clarke have no conflicts of interest to declare.
